# Impaired right ventricular ejection fraction after cardiac surgery is associated with a complicated ICU stay

**DOI:** 10.1186/s40560-018-0351-3

**Published:** 2018-12-27

**Authors:** Inge T. Bootsma, Thomas W. L. Scheeren, Fellery de Lange, Johannes Haenen, Piet W. Boonstra, E. Christaan Boerma

**Affiliations:** 1 Department of Intensive Care, Medical Centre Leeuwarden, Henri Dunantweg 2, P.O. Box 888, 8901 Leeuwarden, the Netherlands; 2 Department of Anaesthesiology, University of Groningen, University Medical Centre Groningen, Groningen, the Netherlands; 3 Department of Cardiothoracic Anaesthesiology, Medical Centre Leeuwarden, Leeuwarden, the Netherlands; 4 Department of Cardiothoracic Surgery, Medical Centre Leeuwarden, Leeuwarden, the Netherlands

**Keywords:** Right ventricle, Thermodilution, Morbidity, Right ventricular function, Cardiac surgery, Intensive care, Pulmonary artery catheter

## Abstract

**Background:**

Right ventricular (RV) dysfunction is a known risk factor for increased mortality in cardiac surgery. However, the association between RV performance and ICU morbidity is largely unknown.

**Methods:**

We performed a single-centre, retrospective study including cardiac surgery patients equipped with a pulmonary artery catheter, enabling continuous right ventricular ejection fraction (RVEF) measurements. Primary endpoint of our study was ICU morbidity (as determined by ICU length of stay, duration of mechanical ventilation, usage of inotropic drugs and fluids, and kidney dysfunction) in relation to RVEF. Patients were divided into three groups according to their RVEF; < 20%, 20–30%, and > 30%.

**Results:**

We included 1109 patients. Patients with a RVEF < 20% had a significantly longer stay in ICU, a longer duration of mechanical ventilation, higher fluid balance, a higher incidence of inotropic drug usage, and more increase in postoperative creatinine levels in comparison to the other subgroups. In a multivariate analysis, RVEF was independently associated with increased ICU length of stay (OR 0.934 CI 0.908–0.961, *p* < 0.001), prolonged duration of mechanical ventilation (OR 0.969, CI 0.942–0.998, *p* = 0.033), usage of inotropic drugs (OR 0.944, CI 0.917–0.971, *p* < 0.001), and increase in creatinine (OR 0.962, CI 0.934–0.991, *p* = 0.011).

**Conclusions:**

A decreased RVEF is independently associated with a complicated ICU stay.

**Electronic supplementary material:**

The online version of this article (10.1186/s40560-018-0351-3) contains supplementary material, which is available to authorized users.

## Background

Nowadays, a set of robust models for the prediction of perioperative mortality in cardiac surgery has been validated [1–3]. However, mortality only reflects the burden of cardiac surgery to a limited extent. Over the years, mortality has decreased despite rising age and complexity [4–6]. A subset of patients successfully survive the perioperative period at the expense of substantial ICU (intensive care unit)-related interventions, length-of-stay (LOS), and morbidity. Both for individual patients and healthcare administrators, it seems relevant to determine the potential risk factors for a complicated post-cardiac surgery course. As of now, it is unclear whether the individual components of established prediction models for perioperative mortality are also relevant for the prediction of ICU morbidity, such as duration of mechanical ventilation, and ICU LOS. In addition, the prediction models at hand do not incorporate right ventricular (RV) performance [7–10]. Nowadays, RV dysfunction has been recognised as an independent risk factor for mortality in coronary artery bypass surgery (CABG) in combination with a poor left ventricular (LV) function [11], in valve surgery [12], congenital heart disease [13], and recently in a heterogeneous group of cardiac surgery patients not selected for well-known risk factors for RV performance [14].

To this end, we aimed to determine the prognostic value of postoperative RV function in relation to ICU morbidity. Subsequently, we investigated the additional value of individual components of the euroSCORE in a multivariate model.

## Methods

### Study population

We performed a single-centre retrospective study in a tertiary teaching hospital with a closed-format ICU. We included all cardiac surgery patients equipped with a pulmonary artery catheter (PAC) and admitted to the intensive care unit in the postoperative phase between January 2011 and January 2015. Exclusion criteria for this retrospective analysis include isolated coronary artery bypass grafting (CABG) with a good LV function. According to our institutional protocol, a large proportion of patients are equipped with a PAC after induction of anaesthesia. The decision to insert a pulmonary artery catheter was protocol driven and was taken at the start of the surgical procedure by the attending cardio-anaesthesiologist. His/her decision is based upon predefined criteria, entirely restricted to the type of surgery. In practice, only patients with an anticipated uncomplicated CABG procedure were not included.

The same cohort of patients has been used in a previous publication to clarify the association between RV performance post cardiac surgery and all-cause long-term mortality [14].

The study was approved by the local ethical and scientific committee and the need for informed consent was waived in accordance with applicable laws.

### Data collection

In case of postoperative PAC monitoring, patients were equipped with a 7.5 F CEDV-Pulmonary Artery Catheter (model 744H, Baxter Healthcare Corporation, Irvine, CA, USA), directly after induction of anaesthesia. During surgery, additional haemodynamic monitoring was performed by transesophageal echocardiograph*y* (TEE). Prior to transfer to the ICU, the PAC was interfaced with a computerised monitoring system (Vigilance II® CCO/SvO_2_/CEDV Monitor, Edwards Lifesciences Corporation, Irvine, CA, USA). The catheter is equipped with a thermal filament positioned 4 cm from the tip of the catheter. This thermal filament generates heat pulses in a random on-off pattern. The change in blood temperature is measured downstream throughout the entire respiratory cycle. Based on a repeating on-off signal, a relaxation waveform can be generated. The right ventricular ejection fraction can be computed by the exponential slope of this waveform curve and the continuous averaged heart rate. The longer it takes for the curve to reach baseline, the lower the ejection fraction. The random on-off pattern is repeated every 60 s and reflecting an average of the measurements taken over the last 5 to 10 min (time averaging) [15]. This catheter enables continuous measurement of the right ventricular ejection fraction (RVEF), cardiac output, and end-diastolic volume index (EDVi) at a sample rate of one per minute. For each patient, all PAC-derived measurements (cardiac index, pulmonary artery pressure (PAP), central venous pressure (CVP), EDVi, mixed venous oxygen saturation (SvO_2_), and RVEF) started directly after ICU admission and were averaged over the first 24 h of ICU admission. Based upon the Gaussian distribution curve of the postoperative RVEF, patients were stratified into three predefined groups: Group 1: RVEF < 20%, group 2: RVEF 20–30%, group 3: RVEF > 30%.

### Postoperative details

All patients were admitted to the ICU direct postoperatively. In our institution, by protocol, all postoperative cardiac surgery patients are admitted to the ICU on mechanical ventilation. No cases were extubated in the operating room.

Settings of mechanical ventilation were standardised according to local protocol, with a respiratory frequency of 20–30 times per minute, tidal volumes limited up to 6 ml/kg ideal bodyweight, and a postoperative end-expiratory pressure (PEEP) of 10 cm H_2_O. Extubation was performed within 2 h of ICU admission in case the patient was haemodynamically stable and in absence of signs of (surgical) complications (bleeding, infarction). All patients were discharged from the ICU on the first postoperative day, unless they had unstable and/or insufficient haemodynamic parameters, need for inotropes or vasopressor, signs of surgical complications (bleeding, infarction), and/or signs of organ failure.

### Statistical analyses

Statistical analysis was performed using the statistical package for social science (SPSS 21 for Windows; SPSS Inc., Chicago, IL, USA). Data are described as median with interquartile range (IQR) unless stated otherwise. Kolmogorov-Smirnov test was used to test normal distribution. Differences between groups were performed with applicable non-parametric tests for unpaired data. Correlations were tested with the Spearman Rho (R_s_) test. Bivariate logistic regression of the association between the RVEF and morbidity was performed using the backward likelihood ratio method. Markers of morbidity were transformed into dichotomous variables by using the 75th percentile as a cut off value.

Baseline characteristics with a *p* value < 0.25 in the univariate analysis were included. For multi-categorical variables, the group with the highest number of patients served as reference category. All statistics were two-tailed and considered statistically significant if the *p* value was < 0.05. All euroSCORE variables with a *p* value < 0.25 in the univariate analysis were incorporated in the multivariate model as separate components.

### Endpoints

The primary endpoint of our study was ICU morbidity (as determined by ICU LOS, duration of mechanical ventilation, usage of inotropic drugs and fluids, and kidney dysfunction) in relation to right ventricular ejection fraction.

Secondary endpoints included the correlation between RVEF and different haemodynamic variables (PAP, cardiac index, EDVi, CVP, and SvO_2_).

## Results

### Baseline characteristics

In the 4-year study period, cardiac surgery was performed in 3094 patients. A total of 1109 patients matched the inclusion criteria. 216 patients were assigned to group 1 (RVEF 18 (15–19)%), 747 patients to group 2 (RVEF 25 (23-27)%), and 146 to group 3 (RVEF 32 (30-34)%). Baseline characteristics are listed in Table 1. The type of operation is further differentiated in (Additional file 1: Table S1). Differences between groups were observed in demographic parameters (age, bodyweight), cardiac status (unstable angina, pre-operative LV performance), and other clinical parameters (APACHE IV and preoperative pulmonary hypertension). In detail, patients with a low RVEF were older; had more often COPD, pulmonary hypertension, or unstable angina pectoris; and had a reduced preoperative left ventricular function and a higher APACHE IV and EuroSCORE. No differences were found between intraoperative characteristics and type of procedure.

**Table 1:** Baseline characteristics

	RVEF < 20% *N* = 216	RVEF 20–30% *N* = 747	RVEF > 30% *N* = 146	*p* value
Demographics				
Age (years)	74 [67–79]	70 [63–77]	66 [58–73]	< 0.001*
Male sex (%)	65	64	69	0.635
Body weight (kg)	76 [68–86]	80 [71–90]	83 [72–93]	0.002*
Comorbidities (%)				
Diabetes mellitus	18	21	16	0.367
Peripheral vessel disease	15	15	17	0.565
TIA/CVA	16	121	12	0.269
Neurological dysfunction	4	4	5	0.772
COPD	22	17	12	0.038*
Cardiac status (%)				
Recent myocardial infarction (< 90 days)	12	12	9	0.602
Previous cardiac surgery	19	22	20	0.618
Unstable angina	4	5	1	0.036*
Preoperative LVEF (%) (TTE)				
- Good (> 55%)	40	59	66	
- Moderate (30–49%)	33	26	24	< 0.001*
- Poor (< 30%)	27	15	10	
Preoperative status				
Preoperative serum creatinine (μmol/l)	88 [76–107]	88 [75–102]	89 [77–100]	0.638
Preoperative pulmonary hypertension (%)	7	3	1	0.013*
Intraoperative characteristics				
Aortic cross-clamp (min)	98 [71–129]	97 [68–140]	93 [64–133]	0.578
ECC (min)	139 [96–187]	137 [99–190]	126 [89–182]	0.363
Procedure (%)				
CABG	13	12	11	0.285
Valve	49	45	54	
CABG + valve	28	32	23	
Other	10	10	12	
Risk scores				
APACHE IV	52 [43–62]	50 [40–59]	46 [36–54]	< 0.001*
EuroSCORE I	8 [6–10]	7 [5–9]	6 [4–8]	< 0.001*

*Indicates a significant difference across groups

*Abbreviations*: APACHE, Acute Physiology and Chronic Health Evaluation; CABG, coronary artery bypass grafting; COPD, chronic obstructive pulmonary disease; CVA, cerebral vascular accident; ECC, extracorporeal circulation; EuroSCORE, European System for Cardiac Operative Risk Evaluation; LVEF, left ventricular ejection fraction; RVEF, right ventricular ejection fraction; TIA, transient ischemic attack; TTE, transthoracic echocardiography

### Primary endpoint

**Fig. 1:**
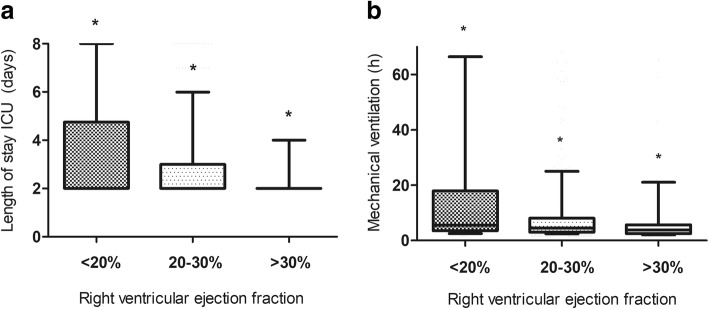
**a** Median and 10–90th percentile of length of stay ICU. **b** Median and 10–90th percentile of duration of mechanical ventilation

In a univariate analysis, patients with the lowest RVEF (< 20%, group 1) had a significantly longer stay in ICU (*p* < 0.001, Fig. 1a) and a longer duration of mechanical ventilation (*p* < 0.001, Fig. 1b) compared to patients with RVEF > 20% Furthermore, there was a significant more positive fluid balance (*p* < 0.001), a higher incidence of inotropic drug usage (*p* < 0.001), and a larger increase in postoperative creatinine (*p* = 0.004) (Table 2). There were no differences in postoperative complications between groups.

**Table 2:** Postoperative details

	RVEF < 20%	RVEF 20–30%	RVEF > 30%	*p* value
Mortality ICU (%)	4.2%	1.2%	0.7%	0.008*
LVEF (%) (TEE)				
Good (> 55%)	52	69	72	
Moderate (30–49%)	31	24	21	< 0.001*
Poor (< 30%)	17	8	9	
Markers of morbidity				
Length of stay ICU (days)	2 [2–5]	2 [2–3]	2 [2–2]	< 0.001*
Mechanical ventilation (h)	5.5 [3.5–17.8]	4.5 [3–8]	3.8 [2.5–5.6]	< 0.001*
Fluid balance (l)	1.9 [1.2–3.2]	1.6 [0.8–2.8]	1.1 [0.3–2.0]	< 0.001*
Use of inotropic drugs (%)	76	61	48	< 0.001*
Postoperative laboratory details				
Delta creatinine (μmol/l)^‡^	12 [− 13–41]	5 [−9–28]	0 [−10–19]	0.004*
Peak lactate (mmol/l)	2.6 [1.9–3.4]	2.7 [2.0–3.5]	2.7 [2.0–3.5]	0.431
Peak CK-MB (U/l)	39 [24–72]	44 [28–76]	40 [27–66]	0.095
Postoperative complications (%)				
Re-sternotomy	7.4	6.6	6.8	0.910
Tamponade	4.6	3.6	4.8	0.686
CVVHD	4.2	1.9	1.4	0.101
Haemodynamic variables†				
RVEF (%)	18 [15–19]	25 [23–27]	32 [31–34]	< 0.001*
Mean PAP (mmHg)	20 [17–25]	19 [15–22]	18 [16–21]	< 0.001*
Cardiac index (l/min/m^2^)	2.2 [2.0–2.5]	2.4 [2.2–2.7]	2.7 [2.4–3.0]	< 0.001*
EDVi (ml/m^2^)	143 [124–167]	113 [100–128]	101 [85–115]	< 0.001*
CVP (mmHg)	8 [5–10]	7 [5–9]	7 [5–9]	0.081
SvO_2_ (%)	64 [59–68]	65 [60–69]	68 [63–71]	< 0.001*
PHT (%)	24	9	7	< 0.001*

^‡^Postoperative delta creatinine is the difference between preoperative creatinine and postoperative peak value creatinine. †Mean values measured with a pulmonary artery catheter over the first 24 h of ICU admission

Abbreviations: ICU, intensive care unit; CK-MB, creatine kinase-myoglobin binding; CVVHD, continuous veno-venous haemodialysis; LV, left ventricular; RVEF, right ventricular ejection fraction; PAP, pulmonary artery pressure; EDVi, end-diastolic volume index; CVP, central venous pressure; SvO_2,_ mixed venous oxygen saturation; PHT, pulmonary hypertension; TEE, transoesophageal echocardiography

After multivariate analysis, RVEF as a continuous variable was independently associated with ICU LOS (OR 0.934 CI 0.908–0.961, *p* < 0.001), duration of mechanical ventilation (OR 0.969, CI 0.942–0.998, *p* = 0.033), the use of inotropic drugs (OR 0.944, CI 0.917–0.971, *p* < 0.001), and increase in creatinine (OR 0.962, CI 0.934–0.991, *p* = 0.011) (Table 3). In addition, we also observed a significant correlation coefficient between CVP and rise in serum creatinine (*r*_s_ = 0.21, *p* < 0.001). The odds ratios are a decreased risk for every increase in RVEF per percentage in model 1, and in model 2, the odds ratios are an increased risk compared to the group with the best RVEF. (> 30%, group 3).

**Table 3:** Binary logistic regression analysis with odds ratios for markers of morbidity

	Lengths of stay ICU ≥ 3 days	Mechanical ventilation ≥ 8.5 h	Fluid balance ≥ 2.8 l	Use of inotropic drugs (Y/N)	Increase in creatinine ≥ 30 μmol/l
Model 1: RVEF as a continuous variable					
RVEF (%)	0.934 [0.908–0.961] *p* < 0.001*	0.969 [0.942–0.998] *p* = 0.033*	0.974 [0.947–1.002] *p* = 0.065	0.944 [0.917–0.971] *p* < 0.001*	0.962 [0.934–0.991] *p* = 0.011*
Age (years)	0.984 [0.969–0.999] *p* = 0.038*	0.988 [0.972–1.004] *p* = 0.143	0.995 [0.979–1.011] *p* = 0.538	0.982 [0.968–0.997] *p* = 0.018*	1.000 [0.982–1.016] *p* = 0.907
Bodyweight (kg)	1.001 [0.991–1.010] *p* = 0.914	1.000 [0.989–1.010] *p* = 0.942	0.998 [0.988–1.009] *p* = 0.760	0.996 [0.986–1.005] *p* = 0.393	1.019 [1.009–1.029] *p* = < 0.001*
COPD	1.192 [0.833–1.706] *p* = 0.336	1.136 [0.777–1.661] *p* = 0.510	1.120 [0.774–1.620] *p* = 0.548	1.110 [0.764–1.613] *p* = 0.584	0.824 [0.557–1.219] *p* = 0.332
Unstable angina	4.599 [2.309–9.163] *p* < 0.001*	4.671 [2.433–8.969] *p* < 0.001*	2.302 [1.237–4.284] *p* = 0.008*	1.538 [0.665–3.552] *p* = 0.314	1.203 [0.615–2355] *p* = 0.589
Poor LVEF^†^	1.385 [0.950–2.020] *p* = 0.091	1.044 [0.737–1.479] *p* = 0.807	0.806 [0.536–1.212] *p* = 0.300	11.579 [6.393–20.973] *p* < 0.001*	1.453 [0.986–2.139] *p* = 0.059
Pulmonary hypertension	1.321 [0.662–2.635] *p* = 0.430	1.187 [0.574–2.453] *p* = 0.644	1.067 [0.516–2.206] *p* = 0.861	2.284 [0.931–5.601] *p* = 0.071	2.179 [1.113–4.268] *p* = 0.023*
APACHE IV score	1.061 [1.049–1.073] *p* < 0.001*	1.057 [1.045–1.068] *p* < 0.001*	1.044 [1.034–1.055] *p* < 0.001*	1.038 [1.027–1.050] *p* < 0.001*	1.050 [1.039–1.061] *p* = < 0.001*
Model 2: RVEF as a categorical variable with RVEF > 30% as reference group					
RVEF 20–30%	2.050 [1.258–3.340] *p* = 0.004*	1.301 [0.794–2.131] *p* = 0.297	1.422 [0.878–2.302] *p* = 0.152	1.559 [1.050–2.316] *p* = 0.028*	1.190 [0.741–1.910] *p* = 0.472
RVEF < 20%	3.250 [1879–5.621] *p* < 0.001*	1.896 [1.093–3.288] *p* = 0.023*	1.732 [1.007–2.977] *p* = 0,047*	2.259 [1.359–3.754] *p* = 0.002*	1.633 [0.949–2.812] *p* = 0.077

* Indicates a significant difference across groups. ^†^ Left ventricular ejection fraction < 30%

*Abbreviations:* CI, confidence interval; RVEF, right ventricular ejection fraction; ICU, intensive care unit; COPD, chronic obstructive pulmonary disease; LVEF, left ventricular ejection fraction; APACHE, Acute Physiology and Chronic Health Evaluation

### Secondary endpoints

In a univariate analysis, patients with the lowest RVEF (< 20%) had a significant higher PAP and EDVi. Furthermore, they had a significant lower cardiac index and SvO_2_ (Table 2).

**Fig. 2:**
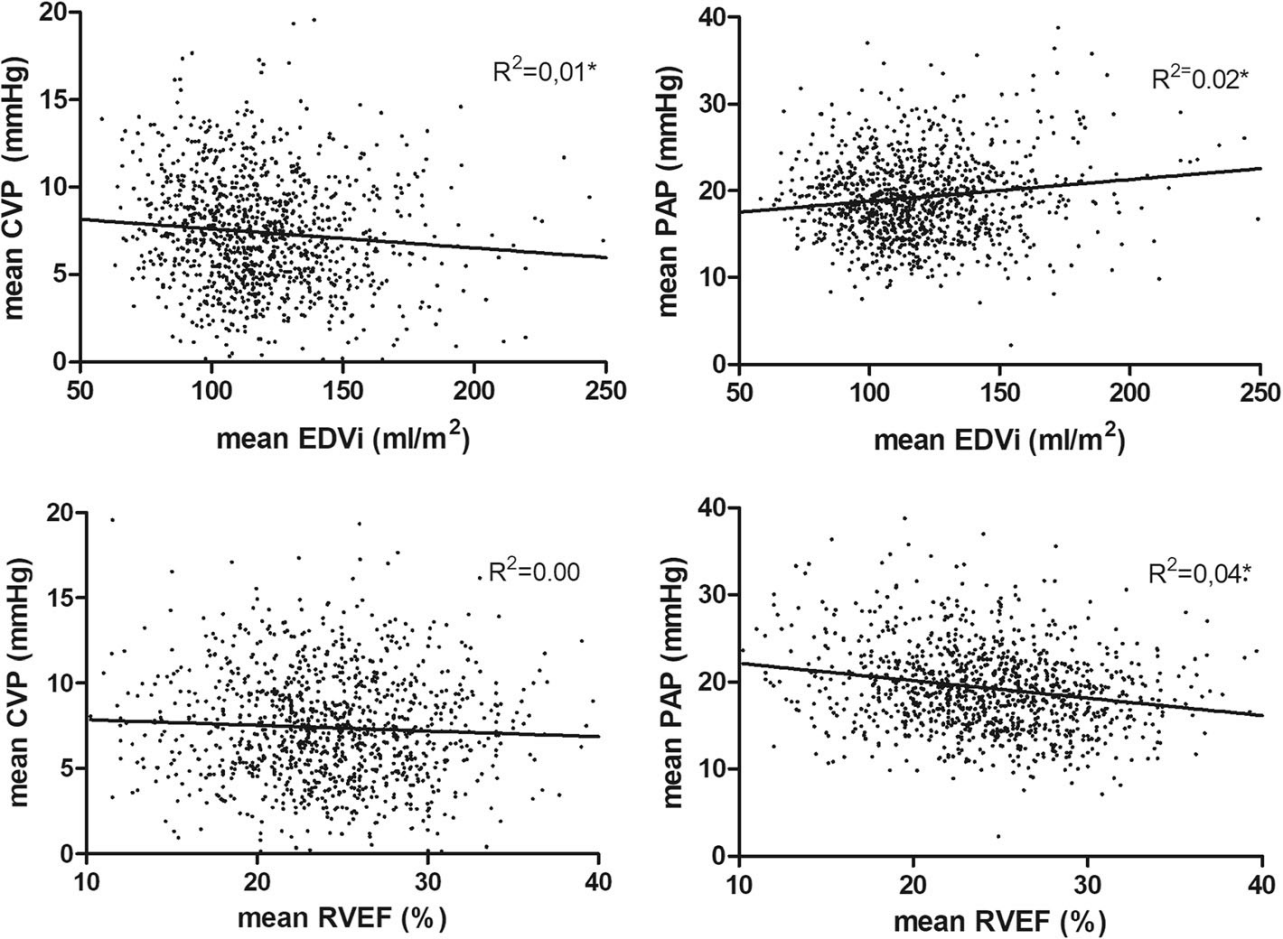
Relation between pressure (CVP or PAP) and volume (EDVi or RVEF)-derived variables. *N* = 1109. Mean values measured with a pulmonary artery catheter over the first 24 h of ICU admission. * indicates a significant correlation between variables. *Abbreviations:* CVP central venous pressure; PAP pulmonary artery pressure; EDVi end-diastolic volume index; RVEF right ventricular ejection fraction

We observed a significant, but weak correlation between the PAP and RVEF (*r*_s_ = − 0.187, *p* < 0.001), CVP and EDVi (*r*_s_ = − 0.135, *p* < 0.001), and PAP and EDVi (*r*_s_ = 0.062, *p* = 0.039). In addition, there was no significant correlation between the CVP and RVEF (*r*_s_ = − 0.052, *p* = 0.084) (Fig. 2).

**Fig. 3:**
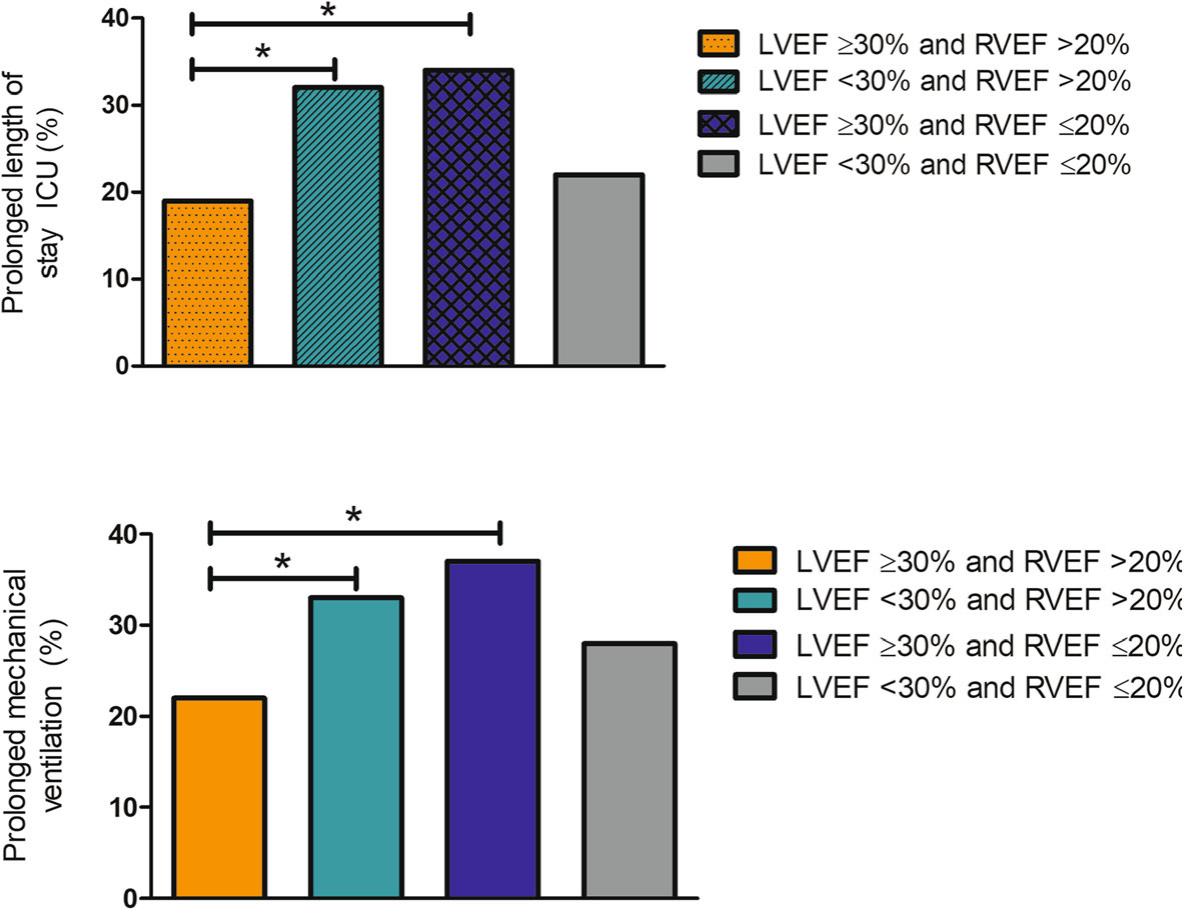
Percentage of patients with a prolonged ICU stay (above) and prolonged mechanical ventilation (below) when separated in 4 different groups according to their LV performance, measured with transthoracic echocardiography preoperatively, and RV performance measured with pulmonary artery catheter postoperatively. * means a statistical difference among groups. Abbreviations: LV left ventricle; RV right ventricle; LVEF left ventricular ejection fraction; RVEF right ventricular ejection fraction; ICU intensive care unit

To further elaborate potential confounding factors in the relationship to RVEF (predominantly, LV performance and pulmonary hypertension), we separated LV and RV performance into four groups: group 1: LVEF ≥30% and RVEF ≥20%, group 2: LVEF <30% and RVEF >20%. group 3: LVEF ≥ 30% and RVEF ≤20%, and group 4: LVEF <30% and RVEF ≤20%. Poor LV function was defined as an EF < 30%, and poor RV function as an EF < 20%. Isolated poor LV performance is statistically associated with the presence of prolonged ICU stay (*p* ≤ 0.001, Fig. 3). In addition, this is also true for those patients with a good LV function and an isolated poor RV performance (*p* < 0.001). Furthermore, both isolated poor LV and RV performance are also statistically associated with prolonged mechanical ventilation (poor LV: *p* < 0.010, poor RV: *p* < 0.001, Fig. 3).

The second main confounder is most likely pulmonary artery pressure (which relates to COPD as well). In addition to the correlation coefficient (Fig. 3), we separated the group according to the international definition of pulmonary hypertension (mean PAP ≥ 25 mmHg) [16] in the first 24 h postoperatively). The percentage of patients with postoperative pulmonary hypertension was significantly different across groups: 7% in patients with RVEF > 30%, 9% in patients with a RVEF, and between 20 and 30% and 24% in patients with a RVEF < 20% (*p* < 0.001).

In addition, 121/216 patient (56%) had a RVEF < 20% in the absence of a poor LV performance or pulmonary hypertension. Interestingly, the percentage of patients with a prolonged length of stay ICU and prolonged mechanical ventilation was not significantly different between those two subgroups.

## Discussion

In this study, we aimed to evaluate the prognostic value of RV performance and to identify risk factors for ICU morbidity. There was a striking absence of association between well-known risk factors for mortality, such as left ventricular function and pulmonary artery pressure, and markers of ICU morbidity. With the exception of unstable angina, all variables of the euroSCORE model did not independently predict ICU LOS or duration of mechanical ventilation in our population. On the other hand, the performance of the RV (which is not included in the euroSCORE or APACHE IV) was independently associated with markers of ICU morbidity. However, it must be stressed that the total package of easily obtainable variables in the euroSCORE and APACHE IV score is a robust and well-validated method to predict outcome.

Over the last decades, mortality in cardiac surgery has decreased despite an increase in age and complexity of surgery [6, 17]. At the same time, there has been an increase in postoperative morbidity, resulting in prolonged ICU stay and an increased use of ICU resources [17, 18]. This increase in ICU morbidity has great impact on the availability of ICU beds, costs, and the burden of suffering for individual patients [19]. Several studies focused on the prediction of the LOS ICU [17, 20, 21]. In contrast to our findings, previous studies mainly identified age, female gender, poor left ventricular function, type of surgery, and renal failure as risk factors for prolonged ICU stay.

In addition, the euroSCORE and Parsonnet score were reported to be suitable as risk scores for ICU morbidity [17, 18, 22]. It is conceivable that we could not reproduce the impact of the individual components of the euroSCORE, due to the limited impact of the separate variables. Moreover, the original validation of the Parsonnet score was performed in a cardiac surgery population with a considerably higher mortality (up to 20%) in comparison to our study [2]. Alternatively, the unique addition of the postoperative RV performance to these otherwise preoperative risk factors may have influenced our predictive model considerably.

Several studies addressed the contribution of RV performance on ICU morbidity and mortality in cardiac surgery. Among patients with hypotension, the majority of the non-survivors appeared to classify as isolated RV failure or biventricular failure. In contradiction, isolated LV dysfunction was associated with a significant better prognosis as compared to RV dysfunction [23, 24]. In CABG patients with a poor LV function, a fractional area change of the RV (RVFAC) ≤ 35% was associated with prolonged duration of mechanical ventilation, ICU and hospital LOS, and increased use of inotropic drugs [11]. Furthermore, RV dysfunction was associated with a high mortality rate in myocardial infarction [25] and valve surgery [12]. Recently, we identified a poor RV performance as a strong independent risk factor for long-term mortality in a large mixed cardiac surgery population [14]. Although the clinical impact of RV performance on mortality seems to be established, robust data on the influence of RV performance after cardiac surgery on ICU morbidity are still lacking [7, 26]. Our data indicate to incorporate RV function into future predictive models.

A possible explanation for the absence of postoperative RV performance in predictive models is the fact that RV dysfunction may well be under-diagnosed. The complex anatomy of the RV makes it challenging to assess its function [27]. Although there are several reliable and reproducible echocardiographic variables to assess RV function, image quality by transthoracic echocardiography (TTE) in the postoperative phase after cardiac surgery is often limited due to mediastinal air, drains, dressings, and the supine position [28–30]. The lowest image quality with TTE has been found on the first postoperative day [31]. Generally, for transesophageal echocardiography (TEE), sedation is required for tolerance, which does not make it a suitable investigation for the awake patient in the postoperative phase. Furthermore, a trained user for TEE/TTE will not be available for 24 h a day and data are based upon single spot measurements. Despite the general debate about the use of PAC, its newest generation enables continuous measurement of the RVEF, EDVi, cardiac index, and SvO_2_ and will overcome the above limitations. Furthermore, our data showed a significant correlation between TEE findings and PAC-derived RVEF. Despite all odds, recent data indicate a benefit of the use of PAC in the setting of cardiac surgery [32, 33].

The lack of clinically relevant correlations between volumetric variables (RVEF/EDVi) and classical pressure variables, such as CVP and PAP, is an important observation in our study. Although an inconvenient truth, such observations are supported by physiological theory. First of all, pulmonary hypertension is a well-known risk factor for RV dysfunction, since it is the main determinant of RV afterload [34]. However, this does not imply that other factors may not contribute to impaired RV function. It is noteworthy that in our study-population median PAP was as low as 20 (17–25) in the first 24 h postoperatively in patients with a RVEF < 20%. Only 24% of the patients with a RVEF < 20% meets the criteria of pulmonary hypertension. These data underline what is known from literature: pulmonary hypertension is a risk factor for RV failure. However, it also implies that a substantial percentage of patients with a low RVEF does not have pulmonary hypertension. And vice versa, pulmonary hypertension does not necessarily result in RV dysfunction [35]. Although sensitive to a sudden increase in afterload, a more gradual increase in pulmonary vascular resistance may result in adequate coping of the RV by increased contractility and remodelling [36].

Pulmonary hypertension may not be the only contributing factor to RV failure in the postoperative setting; LV dysfunction, ischemia, and positive-pressure ventilation have been associated with RV performance as well [25, 37, 38]. We separated our data into four groups according to their left and right ventricular performance. These data underline the idea that there is a subcategory of patients with an isolated poor RV performance (in the absence of a poor LV performance) that has similar prognosis as patients with an isolated poor LV performance (in the absence of a poor RV performance). Although RV and LV performance are clearly related to one another, isolated forms of RV or LV dysfunction seem to exist and have similar prognostic value. The fact that a combined poor LV and RV function did not seem to contribute to impair outcome is at first glance a somewhat unexpected finding. The simple explanation could be that the sample size of the group is apparently too small to detect differences whatsoever. But it is also conceivable that this subgroup may represent a selection bias. Since a poor LV and RV function is likely to be acknowledge preoperatively, surgery may only be performed in the best subgroup of these patients. Alternatively, the combination of a low LV and RV function is a marker of a slowly progressive form of heart failure which allows for adaptation, whereas isolated right heart failure may reflect a more acute form of unadapted heart failure. In a previous report, additional surgery in ischemic heart disease to redress the size of the left ventricle was associated with a reduction in right ventricular function and subsequently a decrease in survival. This data underlines the importance of right and left ventricular remodelling in the setting of heart failure [39, 40].

In addition, CVP may not serve as a surrogate for RV performance either, reflecting the non-linear end-diastolic pressure-volume relation [41]. At low end-diastolic volumes, RV pressure increases minimally for a given increase in volume. At high end-diastolic volumes, the pressure rises disproportionately for a similar increase in volume. This lack of association between CVP and RVEF was illustrated by a correlation coefficient of 0.052 (*p* = 0.084).

Lastly, we observed an independent association between RVEF and a postoperative increase in creatinine. In addition, we also observed a significant correlation between rise in creatinine and CVP. This is in line with the current literature. Venous congestion has been reported to be the strongest haemodynamic determinant for worsening of renal function, independent of cardiac output [42]. The current hypothesis to explain this phenomenon is that an increase in venous pressure serves as an outflow obstruction for organ perfusion [43].

Our study has several limitations. Although the PAC used in our study allows for continuous assessment of RV performance, its use for this purpose may be a topic of debate. Alternatively, RV function may be determined by TEE-derived variables such as Tricuspid Annular Plane Systolic Excursion (TAPSE) and S′ [44, 45]. However, although robust in the prediction of cardiac surgery-related mortality, the prognostic value of these variables for ICU morbidity remains to be established. The retrospective design of our study does not allow for the elaboration of cause-effect relationships. Although we built a multivariate model with a large variety of well-known risk factors, it is conceivable that a yet unknown factor may have served as a potential bias. In order to establish the clinical relevance of the prognostic value of RV function in relation to ICU morbidity, new models that include RV function have to be tested prospectively. Lastly, due to lack of pre-operative values of RV function, this study does not allow to differentiate patients with pre-existing impairment of RV function and those who develop RV dysfunction during cardiac surgery. Future studies are needed to elaborate this important topic.

## Conclusion

In our dataset, RVEF and unstable angina appeared to be independent risk factors for markers of ICU morbidity, such as length of ICU stay, duration of mechanical ventilation, and increased use of inotropic drugs. There was a striking absence of prognostic value for the individual components of the euroSCORE in relation to ICU morbidity. CVP and PAP cannot be used as surrogates for volumetric RV variables. Selection of high-risk cardiac surgery patients in terms of individual burden, costs, and use of ICU resources warrants further research in this field.

## Additional file

Additional file 1: **Table S1.** Operation type. (DOCX 13 kb)

## Abbreviations

APACHE: Acute Physiology and Chronic Health Evaluation
CABG: Coronary artery bypass grafting
CI: Confidence interval
CK-MB: Creatine kinase-myoglobin binding
CO: Cardiac output
COPD: Chronic obstructive pulmonary disease
CVA: Cerebral vascular accident
CVP: Central venous pressure
CVVHD: Continuous veno-venous haemodialysis
ECC: Extracorporeal circulation
EDVi: End-diastolic volume index
EuroSCORE: European System for Cardiac Operative Risk Evaluation
ICU: Intensive care unit
IQR: Interquartile range
LOS: Length of stay
LV: Left ventricular
LVEF: Left ventricular ejection fraction
PAC: Pulmonary artery catheter
PAP: Pulmonary artery pressure
PEEP: Postoperative end-expiratory pressure
RV: Right ventricular
RVEF: Right ventricular ejection fraction
RVFAC: Fractional area change
SvO_2_: Mixed venous oxygen saturation
TAPSE: Tricuspid annular plane systolic excursion
TEE: Transesophageal echocardiography
TIA: Transient ischemic attack
TTE: Transthoracic echocardiography

## References

[1] Nashef SA, Roques F, Michel P, Gauducheau E, Lemeshow S, Salamon R (1999). European system for cardiac operative risk evaluation (EuroSCORE). Eur J Cardiothorac Surg.

[2] Parsonnet V, Dean D, Bernstein AD (1989). A method of uniform stratification of risk for evaluating the results of surgery in acquired adult heart disease. Circulation.

[3] Zimmerman JE, Kramer AA, McNair DS, Malila FM (2006). Acute physiology and chronic health evaluation (APACHE) IV: hospital mortality assessment for today's critically ill patients. Crit Care Med.

[4] Hawkes AL, Nowak M, Bidstrup B, Speare R (2006). Outcomes of coronary artery bypass graft surgery. Vasc Health Risk Manag.

[5] Wang W, Bagshaw SM, Norris CM, Zibdawi R, Zibdawi M, MacArthur R (2014). Association between older age and outcome after cardiac surgery: a population-based cohort study. J Cardiothorac Surg.

[6] Ferguson TB, Hammill BG, Peterson ED, DeLong ER, Grover FL, STS National Database Committee (2002). A decade of change--risk profiles and outcomes for isolated coronary artery bypass grafting procedures, 1990-1999: a report from the STS National Database Committee and the Duke Clinical Research Institute. Society of Thoracic Surgeons. Ann Thorac Surg.

[7] Sanders J, Keogh BE, Van der Meulen J, Browne JP, Treasure T, Mythen MG (2012). The development of a postoperative morbidity score to assess total morbidity burden after cardiac surgery. J Clin Epidemiol.

[8] Ghotkar SV, Grayson AD, Fabri BM, Dihmis WC, Pullan DM (2006). Preoperative calculation of risk for prolonged intensive care unit stay following coronary artery bypass grafting. J Cardiothorac Surg.

[9] Pitkänen O, Niskanen M, Rehnberg S, Hippeläinen M, Hynynen M (2000). Intra-institutional prediction of outcome after cardiac surgery: comparison between a locally derived model and the EuroSCORE. Eur J Cardiothorac Surg.

[10] Huijskes RVHP, Rosseel PMJ, Tijssen JGP (2003). Outcome prediction in coronary artery bypass grafting and valve surgery in the Netherlands: development of the Amphiascore and its comparison with the Euroscore. Eur J Cardiothorac Surg.

[11] Maslow AD, Regan MM, Panzica P, Heindel S, Mashikian J, Comunale ME (2002). Precardiopulmonary bypass right ventricular function is associated with poor outcome after coronary artery bypass grafting in patients with severe left ventricular systolic dysfunction. Anesth Analg.

[12] Wencker D, Borer JS, Hochreiter C, Devereux RB, Roman MJ, Kligfield P (2000). Preoperative predictors of late postoperative outcome among patients with nonischemic mitral regurgitation with ‘high risk’ descriptors and comparison with unoperated patients. Cardiology.

[13] Schuuring MJ, Bolmers PPM, Mulder BJM, de Bruin-Bon RACM, Koolbergen DR, Hazekamp MG (2012). Right ventricular function declines after cardiac surgery in adult patients with congenital heart disease. Int J Cardiovasc Imaging.

[14] Bootsma IT, de Lange F, Koopmans M, Haenen J, Boonstra PW, Symersky T (2017). Right ventricular function after cardiac surgery is a strong independent predictor for Long-term mortality. J Cardiothorac Vasc Anesth.

[15] Leeper B (2003). Monitoring right ventricular volumes: a paradigm shift. AACN Clin Issues.

[16] Galiè N, Humbert M, Vachiery JL, Gibbs S, Lang I, Torbicki A (2016). 2015 ESC/ERS guidelines for the diagnosis and treatment of pulmonary hypertension: the joint task force for the diagnosis and treatment of pulmonary hypertension of the European Society of Cardiology (ESC) and the European Respiratory Society (ERS): endorsed by: Association for European Paediatric and Congenital Cardiology (AEPC), International Society for Heart and Lung Transplantation (ISHLT). Eur Heart J.

[17] Ettema RGA, Peelen LM, Schuurmans MJ, Nierich AP, Kalkman CJ, Moons KGM (2010). Prediction models for prolonged intensive care unit stay after cardiac surgery: systematic review and validation study. Circulation.

[18] Nilsson J, Algotsson L, Höglund P, Lührs C, Brandt J (2004). EuroSCORE predicts intensive care unit stay and costs of open heart surgery. Ann Thorac Surg.

[19] Bhamidipati CM, LaPar DJ, Fonner E, Kern JA, Kron IL, Ailawadi G (2011). Outcomes and cost of cardiac surgery in octogenarians is related to type of operation: a multiinstitutional analysis. Ann Thorac Surg.

[20] Almashrafi A, Elmontsri M, Aylin P (2016). Systematic review of factors influencing length of stay in ICU after adult cardiac surgery. BMC Health Serv Res.

[21] Atoui R, Ma F, Langlois Y, Morin JF (2008). Risk factors for prolonged stay in the intensive care unit and on the ward after cardiac surgery. J Card Surg.

[22] Lawrence DR, Valencia O, Smith EE, Murday A, Treasure T (2000). Parsonnet score is a good predictor of the duration of intensive care unit stay following cardiac surgery. Heart.

[23] Reichert CL, Visser CA, van den Brink RB, Koolen JJ, van Wezel HB, Moulijn AC (1992). Prognostic value of biventricular function in hypotensive patients after cardiac surgery as assessed by transesophageal echocardiography. J Cardiothorac Vasc Anesth.

[24] Costachescu T, Denault A, Guimond JG, Couture P, Carignan S, Sheridan P (2002). The hemodynamically unstable patient in the intensive care unit: hemodynamic vs. transesophageal echocardiographic monitoring. Crit Care Med.

[25] Zehender M, Kasper W, Kauder E, Schönthaler M, Geibel A, Olschewski M (1993). Right ventricular infarction as an independent predictor of prognosis after acute inferior myocardial infarction. N Engl J Med.

[26] Kapadohos T, Angelopoulos E, Vasileiadis I, Nanas S, Kotanidou A, Karabinis A (2017). Determinants of prolonged intensive care unit stay in patients after cardiac surgery: a prospective observational study. J Thorac Dis.

[27] Haddad F, Hunt SA, Rosenthal DN, Murphy DJ (2008). Right ventricular function in cardiovascular disease, part I: anatomy, physiology, aging, and functional assessment of the right ventricle. Circulation.

[28] Hahn RT, Abraham T, Adams MS, Bruce CJ, Glas KE, Lang RM (2014). Guidelines for performing a comprehensive transesophageal echocardiographic examination: recommendations from the American Society of Echocardiography and the Society of Cardiovascular Anesthesiologists. Anesth Analg.

[29] Kasper J, Bolliger D, Skarvan K, Buser P, Filipovic M, Seeberger MD (2012). Additional cross-sectional transesophageal echocardiography views improve perioperative right heart assessment. Anesthesiology.

[30] Rudski LG, Lai WW, Afilalo J, Hua L, Handschumacher MD, Chandrasekaran K (2010). Guidelines for the echocardiographic assessment of the right heart in adults: a report from the American Society of Echocardiography endorsed by the European Association of Echocardiography, a registered branch of the European Society of Cardiology, and the Canadian Society of Echocardiography. J Am Soc Echocardiogr.

[31] Canty DJ, Heiberg J, Tan JA, Yang Y, Royse AG, Royse CF (2017). Assessment of image quality of repeated limited transthoracic echocardiography after cardiac surgery. J Cardiothorac Vasc Anesth.

[32] Brovman EY, Gabriel RA, Dutton RP, Urman RD (2016). Pulmonary artery catheter use during cardiac surgery in the United States, 2010 to 2014. J Cardiothorac Vasc Anesth.

[33] Rajaram SS, Desai NK, Kalra A, Gajera M, Cavanaugh SK, Brampton W (2013). Pulmonary artery catheters for adult patients in intensive care. Cochrane Database Syst Rev.

[34] Pinsky MR (2016). The right ventricle: interaction with the pulmonary circulation. Crit Care.

[35] Bogaard HJ, Natarajan R, Henderson SC, Long CS, Kraskauskas D, Smithson L (2009). Chronic pulmonary artery pressure elevation is insufficient to explain right heart failure. Circulation.

[36] Naeije R, Brimioulle S, Dewachter L (2014). Biomechanics of the right ventricle in health and disease (2013 Grover conference series). Pulmonary circulation.

[37] Santamore WP, Dell'Italia LJ (1998). Ventricular interdependence: significant left ventricular contributions to right ventricular systolic function. Prog Cardiovasc Dis.

[38] Luecke T, Pelosi P (2005). Clinical review: positive end-expiratory pressure and cardiac output. Crit Care.

[39] Couperus LE, Delgado V, Palmen M, van Vessem ME, Braun J, Fiocco M (2017). Right ventricular dysfunction affects survival after surgical left ventricular restoration. J Thorac Cardiovasc Surg.

[40] Kukulski T, She L, Racine N, Gradinac S, Panza JA, Velazquez EJ (2015). Implication of right ventricular dysfunction on long-term outcome in patients with ischemic cardiomyopathy undergoing coronary artery bypass grafting with or without surgical ventricular reconstruction. J Thorac Cardiovasc Surg.

[41] Maughan WL, Shoukas AA, Sagawa K, Weisfeldt ML (1979). Instantaneous pressure-volume relationship of the canine right ventricle. Circ Res.

[42] Mullens W, Abrahams Z, Francis GS, Sokos G, Taylor DO, Starling RC (2009). Importance of venous congestion for worsening of renal function in advanced decompensated heart failure. J Am Coll Cardiol.

[43] Vellinga NA, Ince C, Boerma EC (2013). Elevated central venous pressure is associated with impairment of microcirculatory blood flow in sepsis: a hypothesis generating post hoc analysis. BMC Anesthesiol.

[44] Skinner H, Kamaruddin H, Mathew T (2017). Tricuspid annular plane systolic excursion: comparing transthoracic to transesophageal echocardiography. J Cardiothorac Vasc Anesth.

[45] David JS, Tousignant CP, Bowry R (2006). Tricuspid annular velocity in patients undergoing cardiac operation using transesophageal echocardiography. J Am Soc Echocardiogr.

