# Supplementary material for: Impaired right ventricular ejection fraction after cardiac surgery is associated with a complicated ICU stay
Source: J Intensive Care. 2018 Dec 27;6:85. doi: 10.1186/s40560-018-0351-3 (PMC6307315; doi:10.1186/s40560-018-0351-3)
Supplement: Supplementary file 1 — Table S1. Operation type. (DOCX 13 kb) [file 40560_2018_351_MOESM1_ESM.docx]

| **Table S1: Operation type** | | | | |
| --- | --- | --- | --- | --- |
|  | RVEF <20%  N=216 | RVEF 20-30%  N=747 | RVEF >30%  N=146 | p-value |
| **Procedure (%)** | | | | |
| CABG | 13 | 12 | 10 | 0.736 |
| AVR | 17 | 22 | 28 | 0,046* |
| MVR/MVP | 7 | 7 | 7 | 0,935 |
| Aortic arch | 5 | 5 | 3 | 0,795 |
| CABG+AVR | 16 | 21 | 16 | 0,126 |
| CABG + MVP/MVR | 6 | 7 | 8 | 0,665 |
| TAVI | 7 | 6 | 8 | 0,589 |
| MitraClip | 7 | 2 | 5 | <0.01* |
| Valve combinations | 7 | 7 | 4 | 0,425 |
| Other | 15 | 11 | 11 | 0,383 |
| CABG, coronary artery bypass grafting; AVR, aortic valve repair; MVR, mitral valve replacement; MVP, Mitral valve repair; TAVI, transcatheter aortic valve replacement. | | | | |
